# The Possible Role of Resource Requirements and Academic Career-Choice Risk on Gender Differences in Publication Rate and Impact

**DOI:** 10.1371/journal.pone.0051332

**Published:** 2012-12-12

**Authors:** Jordi Duch, Xiao Han T. Zeng, Marta Sales-Pardo, Filippo Radicchi, Shayna Otis, Teresa K. Woodruff, Luís A. Nunes Amaral

**Affiliations:** 1 Department of Chemical and Biological Engineering, Northwestern University, Evanston, Illinois, United States of America; 2 Departament d’Enginyeria Informàtica i Matemàtiques, Universitat Rovira i Virgili, Tarragona, Spain; 3 Departament d’Enginyeria Química, Universitat Rovira i Virgili, Tarragona, Spain; 4 Howard Hughes Medical Institute, Northwestern University, Evanston, Illinois, United States of America; 5 Department of Obstetrics & Gynecology, Feinberg School of Medicine, Northwestern University, Chicago, Illinois, United States of America; 6 Institute for Women’s Health Research, Northwestern University, Chicago, Illinois, United States of America; 7 Northwestern Institute on Complex Systems, Northwestern University, Evanston, Illinois, United States of America; University of Maribor, Slovenia

## Abstract

Many studies demonstrate that there is still a significant gender bias, especially at higher career levels, in many areas including science, technology, engineering, and mathematics (STEM). We investigated field-dependent, gender-specific effects of the selective pressures individuals experience as they pursue a career in academia within seven STEM disciplines. We built a unique database that comprises 437,787 publications authored by 4,292 faculty members at top United States research universities. Our analyses reveal that gender differences in publication rate and impact are discipline-specific. Our results also support two hypotheses. First, the widely-reported lower publication rates of female faculty are correlated with the amount of research resources typically needed in the discipline considered, and thus may be explained by the lower level of institutional support historically received by females. Second, in disciplines where pursuing an academic position incurs greater career risk, female faculty tend to have a greater fraction of higher impact publications than males. Our findings have significant, field-specific, policy implications for achieving diversity at the faculty level within the STEM disciplines.

## Introduction

The proportion of women faculty members in many STEM fields has been steadily increasing, but at the level of associate and full professor, men continue to far outnumber women [1]. This is troubling because studies suggest that a lack of women in leadership positions has a negative impact on women’s aspirations and advancement [2], [3] and may perpetrate gender biases [4]. Many mechanisms have been proposed to explain the gradual loss of women along the STEM academic career path [5]. For example, Carnes et al. [2] suggested that female faculty in academic medical centers experience a number of systemic and selective pressures that put them at a disadvantage at each step of their pursuit of tenure, and in achieving positions of leadership. These pressures could amount to a “glass ceiling” preventing women’s advancement. Others have referred to the Matthew [6] and Matilda [7] effects as the cause of gender differences, that is, the greater resources awarded to men enable them to further advance their careers beyond what is possible for women.

In contrast, to these concerns, Etzkowitz and Ranga [8] recently suggested that the low number of females in academic positions within STEM disciplines should not be a cause for concern because women do not drop from STEM pursuits when they abandon academic careers but merely pursue STEM careers in other arenas. Curiously, Etzkowitz and Ranga’s “vanish box” perspective [8] does not address whether the reasons for women leaving academia do not detract from a level-playing field or whether women have the opportunity to rise to positions of prominence in non-academic careers.

**Figure 1:**
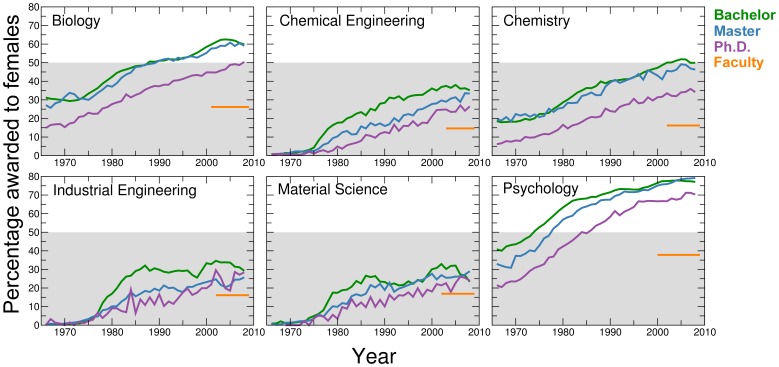
The leaking pipeline. Percentage awarded to females of the total number of bachelor (green lines), master (blue lines) and doctoral (purple lines) degrees in the period 1966–2008. We obtained these data from [40]. We also show the percentage of female faculty in our datasets (orange lines). We could not obtain separate data for molecular biology, so we show the data for biology instead. The grey shaded areas indicate values lower than 50%. The gender ratio of the faculty members given by our data is close to that reported elsewhere. For example, in our data, the percentage of female faculty members in chemistry is 16.2%, and according to the report of Chemical & Engineering News, this percentage is 17% [43].

To determine how and why gender may affect the professional practices and scientific production of researchers, we investigated for seven STEM fields in a quantitative manner the gender-specific and discipline-specific effects of (i) research resource requirements and (ii) relative risk in pursuing an academic career. We explicitly separated the researchers in our database along disciplinary lines in order to more carefully investigate the mechanisms potentially responsible for the observed differences. In contrast to most studies concerned with this matter, we did not conduct surveys but instead systematically analyzed the complete publication records of faculty at a large number of departments in selected research universities in the United States (Table 1, Fig. 1 and Table S4, S5, S6, S7, S8, S9, S10). These data enabled us to characterize the career-long scientific production of a sizable sample of faculty from seven disciplines, and to measure statistically significant differences that would have otherwise remained hidden.

**Table 1:** Female and male cohorts in study.

Discipline	Departments	Female		Male	
		Authors	Publications	Authors	Publications
Chemical Engineering	31	98	6,392	567	66,328
Chemistry	35	198	13,790	1,020	137,723
Ecology	15	106	3,976	328	22,425
Industrial Engineering	15	51	1,498	261	11,509
Material Science	26	98	9,538	473	75,373
Molecular Biology	11	168	9,882	474	51,234
Psychology	10	171	7,143	279	20,976
**Total**		890	52,219	3,402	385,568

### Data

**Figure 2:**
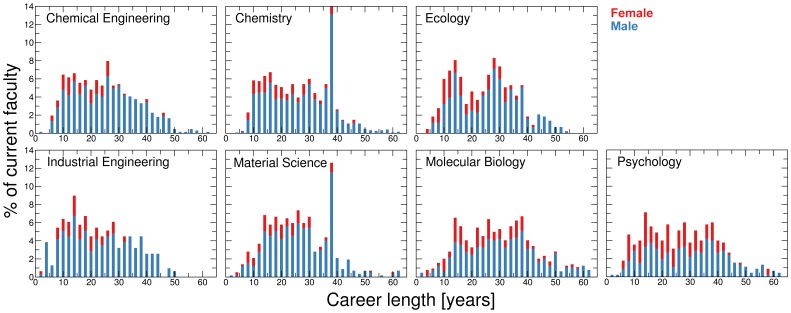
Career lengths of faculty members. Career length distribution of female (red) and male (blue) current faculty members for a selected set of U.S. universities (Table 1). Data is binned into two year intervals. Currently, females hold about 16% of faculty positions in chemistry and in material science departments, and about 25% of faculty positions in molecular biology departments.

We collected data on the 2010 faculty rosters of selected top research institutions in the U.S. (see Supporting Information S1) in seven STEM disciplines – chemical engineering, chemistry, ecology, industrial engineering, material science, molecular biology and psychology – and measured scientific productivity and impact during the various phases of each faculty member’s academic career [9]. We focus on faculty at top U.S. research university departments because most high impact research produced by U.S. authors is published by authors in the top departments. We chose these disciplines for three sets of reasons. First, for all seven disciplines, women only began to join faculty rosters in a consistent manner in the 1980’s, and today they still comprise a small fraction of total faculty (Fig. 1 and Fig. 2). Second, these disciplines cover a broad range of scientific approaches: some place greater emphasis on theoretical or computational work, whereas others focus on industrial applications or on biological systems. Thus the requirement for institutional support – be it lab space [10]–[13], size of start-up packages[10]–[13], or the ability to lead center-level projects – required for success differs dramatically across these disciplines (Table 2). Third, these disciplines pose quite different relative risk profiles to individuals wishing to pursue an academic career. For example, the seven disciplines differ significantly in the prospective earnings of different career options available to Ph.D. graduates and on the time needed to achieve career stability within academia (Table 2).

**Table 2:** Requirement for research resources and risk of academic career choice.

Discipline	Avg. annual expeditureper PI [M$]	Median of salary [K$]		Salary premiumof non-academiccareers, *P^−^* ^1^	Time to career independence, *T*	Frac. of graduates pursuing acad. careers, *A*
		Acad.	Non-acad.			
Chemical Engineering	0.490	77.2	107.2	0.39	5.4	0.21
Chemistry	0.515	64.6	104.2	0.61	6.2	0.32
Ecology	–	69.0	95.0	0.38	8.2	0.71
Industrial Engineering	0.094	74.0	104.0	0.41	6.1	0.56
Material Science	0.612	74.5	102.5	0.38	6.6	0.20
Molecular Biology	1.897	62.2	100.9	0.62	7.3	0.57
Psychology	0.256	65.6	96.2	0.47	8.2	0.42

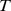
, time to reach career independence. 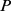, reciprocal of the salary premium of non-academic careers. 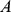, ratio of Ph.D. graduates pursuing an academic position. The data of research expenditures are obtained from http://www.nsf.gov/statistics/nsf11313/. The salary data are obtained from http://www.nsf.gov/statistics/nsf09317/. We use the salary data of zoology instead of ecology because we could not obtain data for ecology. The data of career choice of Ph.D. graduates are obtained from http://www.nsf.gov/statistics/nsf03310/.

## Results

We first focus on research resource requirements. As mentioned earlier, the typical annual research expenditures per faculty member differ substantially across the seven disciplines. For example, industrial engineering faculty tend, for the most part, to train a small number of students at a time. Additionally, much of the research in industrial engineering is theoretical or computational in nature. These two characteristics suggest that, for industrial engineering, researchers do not need to compete against each other for limited resources, and institutional support may not be as important a factor in faculty productivity.

In contrast, most faculty in molecular biology conduct experimental research, and many require significant lab space and expensive specialized equipment. Moreover, faculty in molecular biology are able to compete for funding supporting the creation of large centers or the acquisition of major equipment. Thus, availability of resources, especially institutionally granted resources or institutional support for securing large grants, can be crucial components of academic success in molecular biology [12]. Furthermore, consistent with the Matthew effect [6], [14]–[16], researchers who have already received more institutional support are able to secure even more research resources.

Since historically female faculty members have received less institutional support and have had less access to research resources [17]–[22], these considerations prompt a question with significant policy implications: Could the differences in resource requirements lead to distinct gender-specific publication patterns across disciplines? In order to answer this question, we systematically investigated gender-specific publication rates for the seven disciplines. Even though several studies report greater publication rates by male authors [23]–[27], we hypothesize that only in disciplines where resource requirements are high and institutional support is vital will female faculty members typically publish fewer papers than their male peers. Thus, we predict that gender differences in publication rate in disciplines such as industrial engineering are going to be quite low. In contrast, we predict that gender differences in publication rate are going to be very significant in molecular biology and similar disciplines.

**Figure 3:**
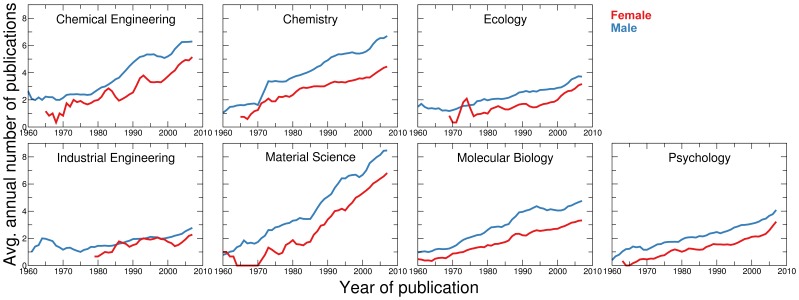
Average number of annual publications per author. Average number of publications authored by females (red) and males (blue) as a function of time. Data is smoothed using moving averaging over a 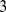-year time window. Note the increasing trend in all disciplines. Because of these trends, we must account for the different starting years and career stages of authors when comparing publication rates.


We define the publication rate of a faculty member 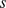 years into her/his career as the number of scientific articles published by the individual 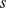 years after her/his first publication. We cannot simply compare the raw publication numbers per year, because these numbers depend strongly on publication year 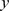 and career stage 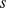 (Fig. 3). Let 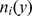 denote the number of publications published by author 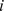 from discipline 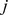 in year 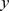, and let 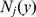 be the total number of authors that have started their careers no later than year 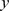. We calculate author 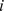
’s z-score (standard score) in year 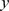 as
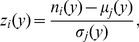
(1)where 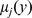 is the average number of publications per author from discipline 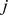 published in year 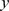


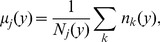
(2)and 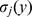 is the standard deviation of the number of papers per author published in year 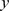


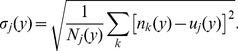
(3)

**Figure 4:**
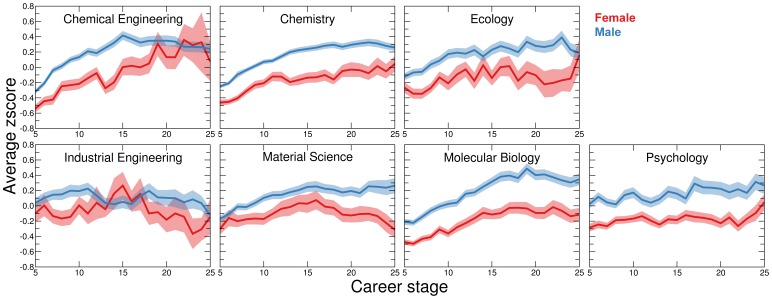
Gender difference in publication rate. Average z-score of number of publications for females (red) and males (blue) as a function of career stage. Shaded areas indicate the standard errors. See Fig. S10 for the statistical significance of the gender difference in publication rate.

In order to account for the effect of career stage, we consider 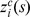, which is the z-score of author 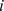 as a function of the career stage 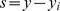, where 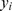 is the year of the first publication of author 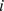 (Fig. 4, S10). Please note that by considering the z-score we are not making any assumption about normality of 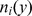, but merely making the results easier to compare across disciplines and time periods.

**Figure 5:**
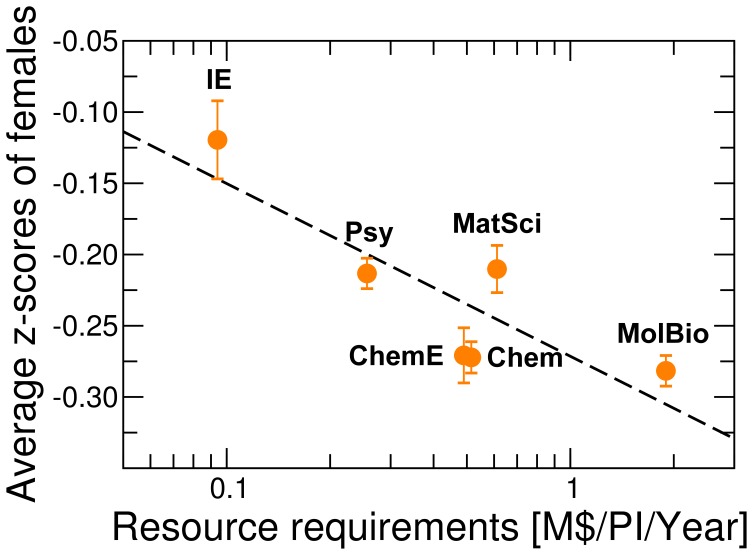
Lower publication rates of female faculty is correlated with higher requirements for research resources. Effects of the magnitude of the resource requirements on the difference in publication rates between genders. Ecology is not included as we could not obtain data for resource requirements. The difference in publication rates is measured by the average z-score of number of publications by females in each year, and the error bars indicate the standard errors. The resource requirements is defined as the average annual research expenditure per principal investigator in the departments studied (Table 2, [40]). The trend line (black dashed line) indicates a negative correlation (coefficient of determination 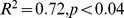). These data suggest that higher resource requirements lead to greater differences in the publication rates between females and their male peers.

Our analysis fully confirms our hypothesis (Fig. 3, 4, 5). As predicted, for disciplines where research expenditures are high, such as molecular biology, we found that females consistently publish at a rate significantly lower than males, whereas for industrial engineering we do not observe a significant difference between genders. More importantly, as shown in Fig. 5, we found that the gender difference in publication rate, measured as the average z-score of females, has a significant negative correlation with magnitude of typical research expenditures. Our results thus support the hypothesis that gender differences in institutional support have had a crucial effect on the publication rates of females.

It is important to point out that in our analysis we did not consider human and social capital such as collaboration level and leadership position, which may also have critical roles for a productive career [4], as research resources. Whether and how the gender difference in the ability to acquire these resources harder to quantify affects career productivity is a matter worth of further investigation.

**Figure 6:**
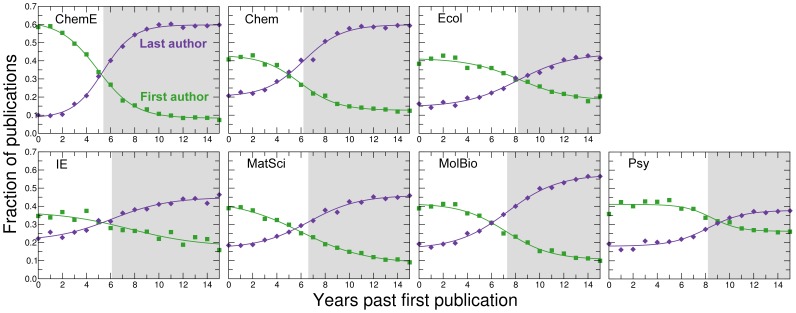
Time to career independence. Fraction of publications in which a faculty member is the last author (purple diamonds) and the fraction of publications in which a faculty member is the first author (green squares). In many disciplines, the senior author of a study is listed last. Looking at the change in the fraction of times a faculty member in our dataset is a first or last author can thus be used as a proxy for change in seniority-level of an individual in these disciplines. We order publications, excluding single-author publications, by years after first publication and aggregate within each discipline. We fit the data to generalized logistic functions (green/purple lines) and define career independence (grey shaded areas) as the mid-point of the logistic function for fraction of last-author publications (Methods, Table S11, S12, S13, S14, S15, S16, S17). While we do not observe gender effects (Figs. S11, S12), we do observe differences between fields.

We next investigated gender-specific and discipline-specific effects of career relative risk profile of an academic career on publication patterns. The risk to pursue a faculty position after obtaining a Ph.D. varies across disciplines. A graduate student considering an academic career in chemistry faces a small risk if unsuccessful. Within about six years from publication of their first paper, successful individuals will move into independent positions (Fig. 6, S11, S12 Table 2 and Methods). Doctoral degree holders in chemistry unable or uninterested in obtaining academic positions can chose from among a number of high-paying careers in industry and government.

In contrast, an individual considering an academic career in ecology faces a much more uncertain future. Instead of waiting six years post publication of the first paper to learn whether it will be possible to secure a faculty position, an ecologist has to wait an average of *eight* years (Fig. 6, S11, S12, Table 2). Perhaps even more challenging, doctoral degree holders in ecology who are not able or not interested in obtaining academic positions may have to settle for jobs that do not pay a significant premium over academic positions.

These observations raise a critical question: Could the different risk profiles of STEM disciplines lead to distinct gender-specific selective pressures? Because pursuing an academic career is a risky undertaking and because propensity towards risk-taking [28], [29], self-motivation towards career development [30], social expectations [31], perception of gender stereotypes [32] and biological constraints [31], [33], [34] are different for females and males, we surmise that a female will choose to pursue an academic career in “high-risk” disciplines, such as ecology, only if she is so highly qualified that she will be quite confident of success. This biased self-selection for outstanding individuals among females likely happens prior to embarking on an academic career [35], leading to females’ advantage in career performance that would be magnified in later stages of career due to the Matthew effect [36]. In contrast, because of the low risk profile of chemistry, we expect that female faculty members in chemistry will incur no extra burden when compared to their male colleagues. It is worth mentioning that an alternative hypothesis is that high career risk induces selection for individuals with greater propensity to risk-taking among females. However, this is consistent with our hypothesis, since risk-taking might be a necessary ingredient, among other intellectual abilities, towards success, and individuals may augment their competence through risk-taking. Therefore, females who enter disciplines with high career risks may be not only risk-takers but in fact also highly qualified.

We further hypothesize that the higher qualification of females in high-risk disciplines will become apparent through higher impact per publication. In order to uncover gender differences in publication impact, we studied a commonly used metric of academic performance, the 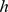-index [37]. We studied the 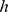-index instead of the total number or average number of citations because the distributions of these numbers can be dramatically biased by a single highly-cited publication [38]. The 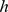-index avoids this bias by identifying the number of publications of an author that have at least that number of citations. Moreover, because the 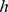-index was introduced after the time period considered for the data, it will not be affected by behaviors of the authors aimed at deliberately increasing their 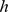-indices.

**Figure 7:**
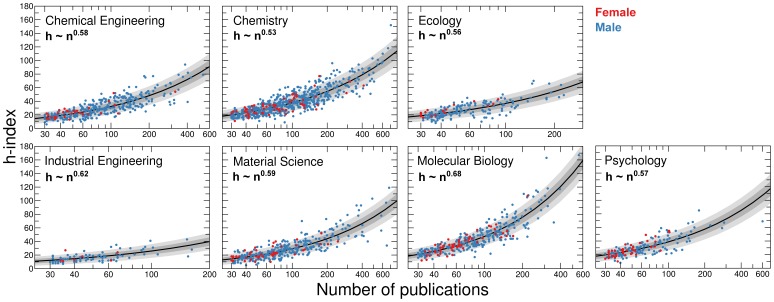
Relation between impact and number of publications. Dependence of the *h*-index on number of publications for faculty with at least 30 publications that are at least 10 years old (Table S18). We consider only publications at least 10 years old in order to ensure that they all have accrued close to their ultimate number of citations [42]. Blue and red dots show values for individual male and female faculty members in our cohorts. The solid black line shows a maximum likelihood power-law fit to 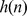 under the assumption of Poissonian fluctuations (Methods). Shaded areas indicate one standard deviation (dark grey areas) and two standard deviations (light grey areas) from the mean.

An identified weakness of the 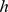-index is its dependence on the number of publications. In order to compare the publication impact of authors with different number of publications, we determined the dependence of the 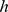-index on the number of publications for the faculty cohorts in the seven disciplines considered. We found that for these seven disciplines the 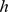-index grows with the number of publications as a power law [39],
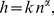
(4)where 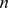 is the number of publications (Fig. 7 and Methods). For 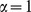, the 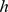-index would grow linearly with number of publications. Importantly, since we find 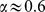, one cannot explain the observed values of 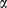 through self-citations alone (Methods).

**Figure 8:**
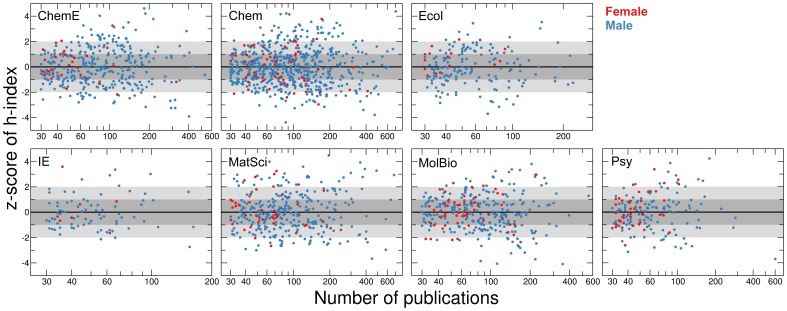
Comparison of publication impact for authors with different numbers of publications. The z-score of the 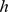-index as a function of number of publications. We use the mean and standard deviation obtained from the parameters in the model to determine the z-scores.

We next measured the deviations of *h*-indices from the trend predicted by Eq. (4) for individual faculty members to obtain the z-scores (standard score) of their publication impact (Fig. 8). Let 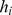 denote the *h*-index of author 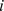, and 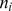 her/his total number of publication. The z-score of *h*-index of author is
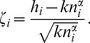
(5)


We then calculated the average z-scores of this publication adjusted 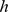-index of females (Fig. 8, S4 and Methods). Our analysis unambiguously shows that for all ranges of number of publications, female faculty members in ecology published research with higher impact than their male counterparts, whereas for faculty in chemistry we found no significant gender-specific differences in impact.

The data in Fig. 8 suggest that the difference 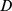 in publication impact may be an increasing function of the discipline-specific risk profile 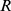 associated with an academic career. That is,
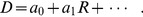
(6)


While we lack a theory for the true definition of career risk, 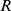, it is plausible that it will be a function of factors such as the time 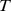 to reach career independence, the fraction 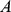 of Ph.D. graduates that go on to careers in academia, and the reciprocal of the salary premium of non-academic careers (Table 2, [40]), which we define as
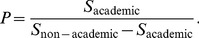
(7)


**Figure 9:**
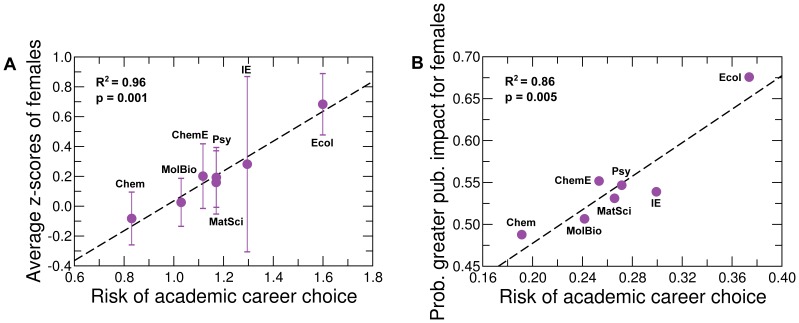
Higher publication impact of female faculty is correlated with higher relative risk of academic career choice. Risk in academic career choice and difference in publication impact. We quantify the risk of academic career choice according to Eq. (10). We show results for two alternative measures of difference in publication impact. In (**A**), we defined the gender difference in publication impact as the average *h*-index z-scores of females. The error bars indicate standard errors. See Fig. S13 for the statistical significance of the gender difference in publication impact. The trend line (black dashed line) indicates a significant positive correlation (coefficient of determination 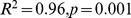). In (**B**), we defined the gender difference in publication impact as the probability that female authors have larger *h*-index z-scores than male authors, as depicted in Fig. S13. The trend line (black dashed line) indicates a significant positive correlation (coefficient of determination 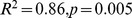). Note that the values of the risk of academic career choice in (**A**) and (**B**) are different for each discipline because the coefficients in the linear regression are different. The data suggest that in disciplines where it is risky to pursue an academic career, female faculty have publications with higher impact than male faculty.

Even though we do not know its functional form, we can expand 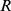 as a multivariate polynomial,
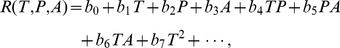
(8)and it follows that we can expand 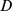 as



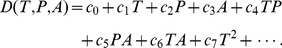
(9)Because we only have 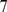 data points, we must fit our data to combinations of at most 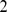 terms in the expansion. Ordinary least squares regression indicates that the difference in publication impact across the seven disciplines is positively correlated with several combinations of the factors in Eq. (9), thus confirming the existence of the relative risk associated with academic careers and its gender-specific role on publication impact (Table 3). In Fig. 9 we show the correlation between the gender difference in publication impact and the academic career risk, quantified as
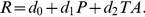
(10)


**Table 3:** Linear models predicting the gender difference in publication impact.

Intercept	*P*	*A*	*TP*	*PA*	*TA*	Adj. *R* ^2^	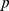 -value
−0.96**	0.39**				0.10**	0.94	0.001
[−1.32, −0.61]	[0.25, 0.54]				[0.06, 0.14]		
−1.03**	0.40**	0.79*				0.88	0.001
[−1.57, −0.50]	[0.19, 0.61]	[0.29, 1.28]					
−0.68*		0.37	0.05*			0.79	0.007
[−1.12, −0.17]		[−0.32, 1.06]	[0.01, 0.08]				
−0.62*			0.05**			0.74	0.007
[−1.14, −0.10]			[0.020 0.09]				
−0.17				0.40*		0.65	0.02
[−0.48, 0.14]				[0.11, 0.69]			
−0.64	0.38					0.42	0.01
[−1.59, 0.32]	[−0.04, 0.79]						

The gender difference in publication impact is defined as the average *h*-index z-scores of females. 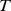, time to reach career independence. 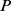, reciprocal of salary premium of non-academic careers. 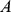, ratio of Ph.D. graduates pursuing an academic position. 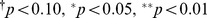. The 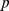-values indicated below were obtained with the permutation test, but using Student’s t-test yields similar results.

This model suggests that in disciplines where there are few non-academic career options available and the time to reach career independence is long, and where it is difficulty to recover salary loss due to unsuccessful academic career, pursuing an academic position is highly risky.

## Discussion

Our study reveals the possible contribution of perceived risk and resource allocation to the under-representation of women in STEM academic careers. Our results are not by themselves an empirical validation of the causal relationship between publication rate and resource requirements, and between publication impact and career risk, since we cannot conduct controlled experiments or account for other factors that could play a role in the measured outcome. However, the hypothesis that there is a causal relationship between gender differences in resource allocation and the reported gender differences in publication rates is plausible and well supported by our empirical observations, as is the hypothesis that there is a causal relationship between the relative risk associated with academic careers and the gender differences in publication impact.

The issues we identify here, together with the known socialization concerns surrounding work-life balance, may have created a “tipping point” that explains the nearly intractable problem of retaining women within STEM disciplines. It is equally important to think about the role these previously unrecognized risk factors may contribute to the number of under-represented minorities in the STEM pipeline. It is not possible to address this point using the methods we describe here, but there may be opportunity and new impetus to develop novel tools that can provide a more sophisticated insight into why some groups of people are not well represented in scientific subspecialties. More intriguingly, we wonder how the perceived or real risks associated with resource infrastructure and future opportunities can be translated into other fields (business, politics, the legal profession) where there is a paucity of women and minorities in the upper career rungs. Most importantly, now that these factors have been identified, it should be possible to create policies that provide better opportunities for all individuals with an aptitude for science, and perhaps in all kinds of careers, to ensure that our work force is diverse and can gain from the insights of all contributing members.

## Methods

### Data Acquisition

We obtained complete faculty rosters as of June, 2010 for several top research universities in the U.S. in the disciplines of chemical engineering, chemistry, ecology, industrial engineering, material science, molecular biology and psychology (see Table S4, S5, S6, S7, S8, S9, S10 for a complete list of institutions and departments that were included in our analysis). We considered all active faculty members, including tenure-track and research faculty, but excluded emeritus professors. For each faculty member, we collected the following data: gender, year of Ph.D. (if available), current and past positions, a list of publications published by the end of 2010 and indexed in Thomson Reuters Web of Science (WoS), and the number of citations for these publications as of June, 2011. To obtain a reliable list of publications for each investigator from the WoS, we designed a supervised disambiguation protocol. Our protocol uses biographic information for an investigator to build and refine a query that retrieves the entire list of publications from the WoS. For example:

1. Select last name and set of initials that the investigator could potentially use to sign her papers. For instance, David A. Tirrell has two potential WoS names, “Tirrell D” and “Tirrell DA.”
2. Set the year of publication range from four years before the Ph.D. date until the data acquisition time. If the Ph.D. year is not available, estimate the Ph.D. year from the list of publications listed in the investigator’s personal web page or from the date of hire. For David A. Tirrell, our protocol returns the publications from 1974 on for “Tirrell D” and “Tirrell DA” (1974 = 1978 - 4 and 1978 is the year Professor Tirrell was awarded a Ph.D.).
3. When current and previous positions are available, constrain the query to retrieve publications that include one of those institutions as one of the author’s address.

The disambiguation protocol downloads all types of publications of the authors. In the analysis we included articles, conference proceedings and reviews. At each step, we obtained the number of publications assigned to a particular author and checked for anomalies using a number of data features, the most important of which were:

1. The total number of publications is consistent with the current position of the investigator, the number of years doing research, and the type of research.
2. The number of publications in each year does not deviate “significantly” from the average of the surrounding years.
3. Journal titles of the publications are within the investigator’s field of expertise.

Our disambiguation protocol allows us to introduce different names or initials for each scientist. For example, for females, for whom there is evidence in the list of publications of their CVs that they change their family name after marriage, we include both names in the query. Note that the errors in the publication list introduced by name changes is small [41]. To estimate the percentage of false positives in the publications assigned to an author, we randomly sampled about one hundred authors in our database who had an updated list of publications on their personal websites. We then manually checked these lists against the results we obtained from the WoS. We estimated that, using our disambiguation protocol, the percentage of false positives in the publications assigned to an author is less than 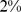.

### Number of Publications and 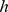-index Distributions

For the analysis of the 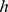-index and the number of publications, we considered only papers published by December 31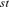, 2000. In order to have a reliable measure of the 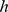-index, we need to consider papers which have accrued a number of citations that truly reflects the impact of that research. Based on prior studies [42], we set ten years as the threshold for papers to have accumulated their “ultimate” number of citations.

### The Value of 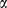 due to Self-citations

Assume that an author with 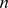 publications makes 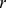 self-citations in each of her/his publications. The total number of self-citations is thus 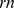. In order to maximize his/her *h*-index, the author will distribute his self-citations homogeneously among 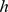 of his own publications. Thus, the average number of citations per publication is 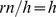, yielding 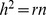 or 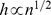. That is, 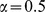.

### Fitting the 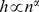 Relationship

We surmise that given the number of publications 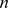, the 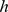-index 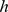 is a random variable obeying the Poisson distribution:
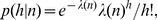
(11)with mean 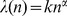. The likelihood of the data given this model is then:
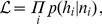
(12)where the product runs over all pairs 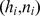 in real data. The best estimates of 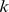 and 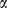 are those that maximize 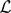. The estimates yield good fits to the data (see Fig. 7).

### Fitting the Transition to Independence

We fitted the data in Fig. 6 to the generalized logistic function,
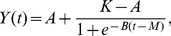
(13)where 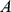 is the lower asymptote, 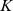 is the upper asymptote, 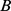 is the growth rate, and 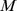 is the time of maximum growth. We provide the values of the fitting parameters for all data sets in Tables S11, S12, S13, S14, S15, S16, S17. We use 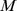 as a proxy for the time for transition to professional independence.

### Statistical Significance of Linear Correlations

The p-values of the linear correlations in Figure 5 and Figure 9 are obtained using two statistical tests, the permutation test and Student’s t-test. Since the Student’s t-test is well known, we describe here only the permutation test. Suppose that we have 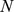 data points on the two dimensional plane. We consider all the 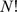 permutations of the 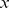 (or 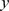) values of the data points, and calculate the correlation coefficient for each of the permutation, which will yield 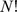 correlation coefficients, 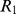, 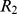, 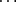, 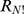. We then calculate the probability that these coefficients are larger than or equal to the correlation coefficient of the original data set 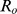, 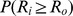. This probability is the p-value given by the permutation test.

## Supporting Information

Figure S1 **Statistical significance of gender difference in publication rate.** Probability that a female faculty member published more articles at a given stage of her career than a male peer at the same career stage (red lines). We use z-scores to account for two trends in the data: (i) the publication rate increases over years (Fig. 3), and (ii) the publication rate varies with the career length (Fig. 4). We indicate the 90% and 95% confidence intervals by the dark grey and light grey areas respectively, and the medians of the probabilities obtained from random ensembles by black lines.
(TIFF)

Figure S2 **Time to career independence of female faculty members.** The fraction of publications authored by female faculty members in which the female faculty member is the last author (red diamonds) and the fraction of publications in which a faculty member is the first author (pink squares). The red/pink lines are fits of the data to a generalized logistic function (Methods, Table S11, S12, S13, S14, S15, S16, S17). The grey shaded areas indicate the periods of professional independence for the different disciplines.
(TIFF)

Figure S3 **Time to career independence of male faculty members.** The fraction of publications authored by male faculty members in which the male faculty member is the last author (blue diamonds) and the fraction of publications in which a faculty member is the first author (azure squares). The blue/azure lines are fits of the data to a generalized logistic function (Methods, Table S11, S12, S13, S14, S15, S16, S17). The grey shaded areas indicate the periods of professional independence for the different disciplines.
(TIFF)

Figure S4
**Statistical significance of gender difference in publication impact.** Probability that female authors have larger *h*-index than male authors when accounting for the number of publications. The red line shows the results for windows including authors with at least 30 publications and at most 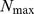 publications. Dark grey areas and light grey areas show the 90% and 95% confidence intervals (see Methods for details).(TIFF)

Table S1 **Gender of faculty in Chemical Engineering departments.**
(PDF)

Table S2 **Gender of faculty in Chemistry departments.**
(PDF)

Table S3 **Gender of faculty in Ecology departments.**
(PDF)

Table S4 **Gender of faculty in Industrial Engineering departments.**
(PDF)

Table S5 **Gender of faculty in Material Science departments.**
(PDF)

Table S6 **Gender of faculty in Molecular Biology departments.**
(PDF)

Table S7 **Gender of faculty in Psychology departments.**
(PDF)

Table S8 **Estimated values of parameters of logistic function for Chemical Engineering data.**
(PDF)

Table S9 **Estimated values of parameters of logistic function for Chemistry data.**
(PDF)

Table S10 **Estimated values of parameters of logistic function for Ecology data.**
(PDF)

Table S11 **Estimated values of parameters of logistic function for Industrial Engineering data.**
(PDF)

Table S12 **Estimated values of parameters of logistic function for Material Science data.**
(PDF)

Table S13 **Estimated values of parameters of logistic function for Molecular Biology data.**
(PDF)

Table S14 **Estimated values of parameters of logistic function for Psychology data.**
(PDF)

Table S15
**Estimated values of parameters of the power law relation between impact and number of publications, 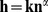.**
(PDF)

Supporting Information S1. (PDF)

## References

[1] Association of American Medical Colleges (2008) Women in U.S. academic medicine statistics and benchmarking report. Technical report.

[2] Carnes M, Morrissey C, Geller SE (2008) Women’s health and women’s leadership in academic medicine: Hitting the same glass ceiling? Journal of Women’s Health 17: 1453–62. 10.1089/jwh.2007.0688 PMC2586600 18954235

[3] Beaman L, Duflo E, Pande R, Topalova P (2012) Female leadership raises aspirations and educational attainment for girls: A policy experiment in India. Science 335: 582–6. 22245740 10.1126/science.1212382 PMC3394179

[4] Petersen AM, Riccaboni M, Stanley HE, Pammolli F (2012) Persistence and uncertainty in the academic career. Proceedings of the National Academy of Sciences 109: 5213–18. 10.1073/pnas.1121429109 PMC3325726 22431620

[5] Ceci SJ, Williams WM (2011) Understanding current causes of women’s underrepresentation in science. Proceedings of the National Academy of Sciences of the United States of America 108: 3157–62. 21300892 10.1073/pnas.1014871108 PMC3044353

[6] Merton RK (1968) The Matthew effect in science. Science 159: 56–63. 5634379

[7] Rossiter M (1993) The Matilda effect in science. Social Studies of Science 23: 325–341.

[8] Etzkowitz H, Ranga M (2011) Gender dynamics in science and technology: From the “leaky pipeline” to the “vanish box”. Brussels Economic Review 54: 131.

[9] US News (2010). URL http://grad-schools.usnews.rankingsandreviews.com/best-graduate-schools.Accessed. July 1, 2010.

[10] University of Pennsylvania Gender Equity Committee (2001) The Gender Equality Report. Technical report, University of Pennsylvania.

[11] CaseWestern Reserve University Equity Study Committee (2003) Resource Equity at CaseWestern Reserve University: Results of Faculty Focus Groups. Technical report, Case Western Reserve University.

[12] Princeton University (2003) Report of the Task Force on the Status of Women Faculty in the Natural Sciences and Engineering at Princeton. Technical report, Princeton University.

[13] National Research Council (2001) Gender Differences at Critical Transitions in the Careers of Science, Engineering, and Mathematics Faculty. Technical report, National Research Council.

[14] Xie Y, Shauman KA (1998) Sex differences in research productivity: New evidence about an old puzzle. American Sociological Review 63: 847.

[15] Prpić K (2002) Gender and productivity differentials in science. Scientometrics 55: 27–58.

[16] Larivière V, Vignola-Gagné E, Villeneuve C, Gélinas P, Gingras Y (2011) Sex differences in research funding, productivity and impact: An analysis of Qúebec university professors. Scientometrics 87: 483–498.

[17] Committee on Women Faculty in the School of Science (1999) A study on the status of women faculty in science at MIT. Technical report, Massachusetts Institute of Technology.

[18] Ginther D (2003) Is MIT an exception? Gender pay differences in academic science. Bulletin of Science, Technology & Society 23: 21–26.

[19] Bornmann L, Daniel H (2007) What do we know about the h index? Journal of the American Society for Information Science and Technology 58: 1381–1385.

[20] Sarfaty S, Kolb D, Barnett R, Szalacha L, Caswell C, et al (2007) Negotiation in academic medicine: A necessary career skill. Journal of Women’s Health 16: 235–44. 10.1089/jwh.2006.0037 17388740

[21] Furnham A, Wilson E (2011) Gender differences in estimated salaries: A UK study. Journal of Socio-Economics 40: 623–630.

[22] Doucet C, Smith M (2012) Pay structure, female representation and the gender pay gap among university professors. Relations Industrielles 67: 51–75.

[23] Cole JR, Zuckerman H (1984) The Productivity Puzzle. In: Maehr ML, Steinkamp MW, editors, Advances in Motivation and Achievement, JAI Press. 217–258.

[24] Long JS (1992) Measures of sex differences in scientific productivity. Social Forces 71: 159.

[25] Xie Y, Shauman KA (2003)Women in Science: Career processes and outcomes. Harvard University Press.

[26] Symonds MRE, Gemmell NJ, Braisher TL, Gorringe KL, Elgar MA (2006) Gender differences in publication output: towards an unbiased metric of research performance. PLoS ONE 1: e127. 17205131 10.1371/journal.pone.0000127 PMC1762413

[27] Jagsi R, Guancial EA, Worobey CC, Henault LE, Chang Y, et al (2006) The “gender gap” in authorship of academic medical literature–a 35-year perspective. The New England Journal of Medicine 355: 281–7. 16855268 10.1056/NEJMsa053910

[28] Byrnes JP, Miller DC (1999) SchaferWD (1999) Gender differences in risk taking: A meta-analysis. Psychological Bulletin 125: 367–383.

[29] Harris C, Jenkins M, Glaser D (2006) Gender differences in risk assessment: Why do women take fewer risks than men. Judgment and Decision Making 1: 48–63.

[30] Cech EA, Blair-loy M (2010) Perceiving glass ceilings? Meritocratic versus structural explanations of gender inequality among women in science and technology. Social Problems 57: 371–397.

[31] Ceci SJ, Williams WM, Barnett SM (2009) Women’s underrepresentation in science: sociocultural and biological considerations. Psychological bulletin 135: 218–61. 19254079 10.1037/a0014412

[32] Heilman M (2001) Description and prescription: How gender stereotypes prevent women’s ascent up the organizational ladder. Journal of Social Issues 57: 657–674.

[33] Mason MA, Goulden M (2002) Do babies matter? The effect of family formation on the lifelong careers of academic men and women. Academe 88: 21–27.

[34] Mason MA (2004) Do babies matter (Part II)? Closing the baby gap. Academe 90: 10–15.

[35] Kaminski D, Geisler C (2012) Survival analysis of faculty retention in science and engineering by gender. Science 335: 864–866. 22344445 10.1126/science.1214844

[36] Petersen AM, Stanley HE, Succi S (2011) Statistical regularities in the rank-citation profile of scientists. Scientific Reports 1: 181. 22355696 10.1038/srep00181 PMC3240955

[37] Hirsch JE (2005) An index to quantify an individual’s scientific research output. Proceedings of the National Academy of Sciences of the United States of America 102: 16569–72. 16275915 10.1073/pnas.0507655102 PMC1283832

[38] Bornmann L, Mutz R, Daniel H (2007) Gender differences in grant peer review: A meta-analysis. Journal of Informetrics 1: 226–238.

[39] Van Raan A (2006) Comparison of the Hirsch-index with standard bibliometric indicators and with peer judgment for 147 chemistry research groups. Scientometrics 67: 491–502.

[40] National Center for Science and Engineering Statistics (NCSES), National Center for Science and Engineering Statistics (2011). URL http://www.nsf.gov/statistics/.Accessed. March 1, 2011.

[41] Radicchi F, Fortunato S, Markines B, Vespignani A (2009) Diffusion of scientific credits and the ranking of scientists. Physical Review E 80: 56103. 10.1103/PhysRevE.80.056103 20365039

[42] Stringer M, Sales-Pardo M, Amaral LAN (2008) Effectiveness of journal ranking schemes as a tool for locating information. PLoS ONE 3: 1–8. 10.1371/journal.pone.0001683 PMC2244807 18301760

[43] Rovner SL (2011) Women Are 17% Of Chemistry Faculty. Chemical & Engineering News 89: 42–46.

