# Correction: The Possible Role of Resource Requirements and Academic Career-Choice Risk on Gender Differences in Publication Rate and Impact

**DOI:** 10.1371/annotation/7f54a3e6-6dcf-4825-9eb9-201253cf1e25

**Published:** 2013-05-17

**Authors:** Jordi Duch, Xiao Han T. Zeng, Marta Sales-Pardo, Filippo Radicchi, Shayna Otis, Teresa K. Woodruff, Luís A. Nunes Amaral

The following information was missing from the Funding section: J. Duch and M. Sales-Pardo’s work have been partially supported by the Spanish DGICYT under project FIS2010-18639. 

