# Supplementary figures and images for: The Possible Role of Resource Requirements and Academic Career-Choice Risk on Gender Differences in Publication Rate and Impact
Source: PLoS One. 2012 Dec 12;7(12):e51332. doi: 10.1371/journal.pone.0051332 (PMC3520933; doi:10.1371/journal.pone.0051332)

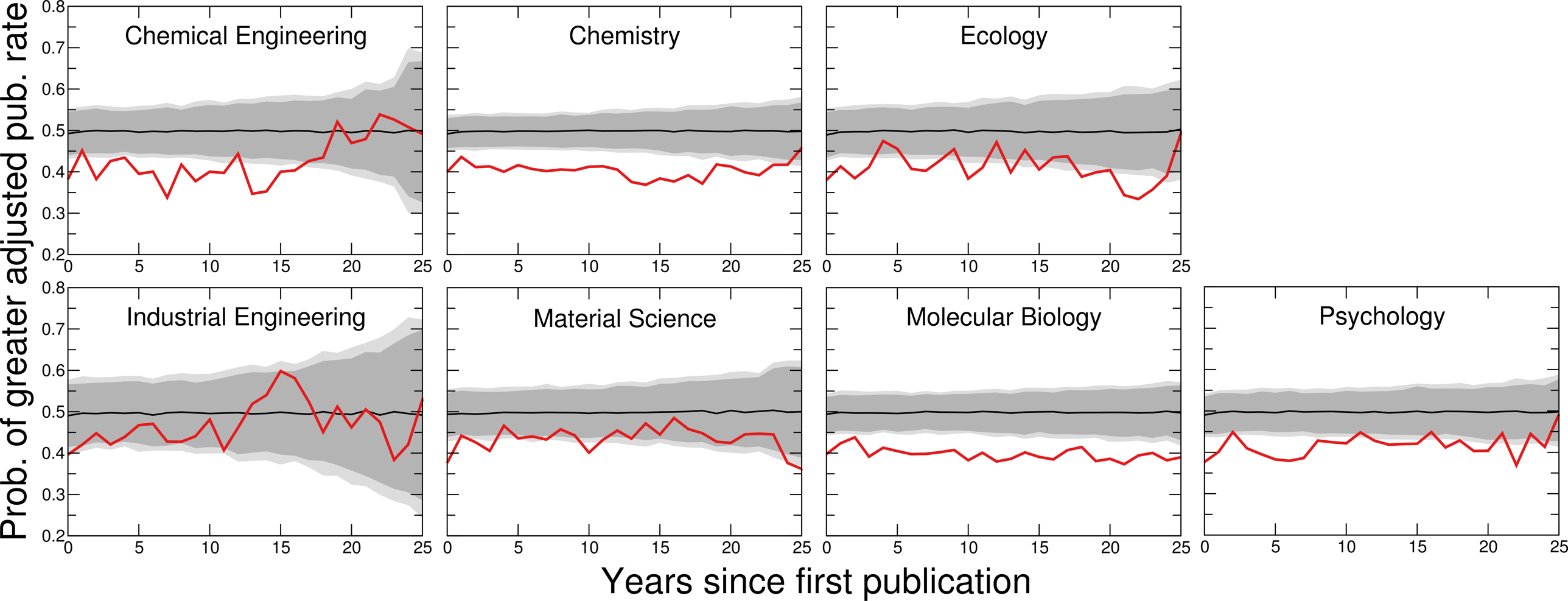

Supplement: Figure S1 — Statistical significance of gender difference in publication rate. Probability that a female faculty member published more articles at a given stage of her career than a male peer at the same career stage (red lines). We use z-scores to account for two trends in the data: (i) the publication rate increases over years (Fig. 3), and (ii) the publication rate varies with the career length (Fig. 4). We indicate the 90% and 95% confidence intervals by the dark grey and light grey areas respectively, and the medians of the probabilities obtained from random ensembles by black lines. (TIFF) [file pone.0051332.s001.tiff]

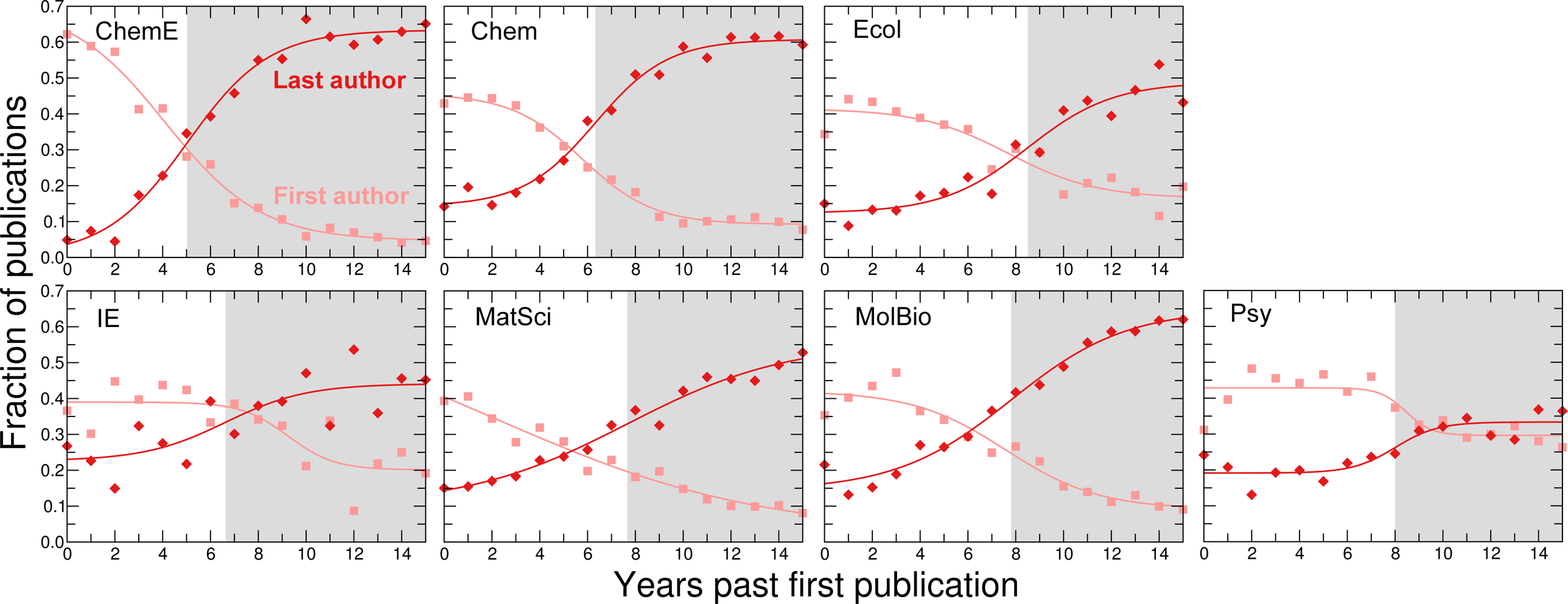

Supplement: Figure S2 — Time to career independence of female faculty members. The fraction of publications authored by female faculty members in which the female faculty member is the last author (red diamonds) and the fraction of publications in which a faculty member is the first author (pink squares). The red/pink lines are fits of the data to a generalized logistic function (Methods, Table S11, S12, S13, S14, S15, S16, S17). The grey shaded areas indicate the periods of professional independence for the different disciplines. (TIFF) [file pone.0051332.s002.tiff]

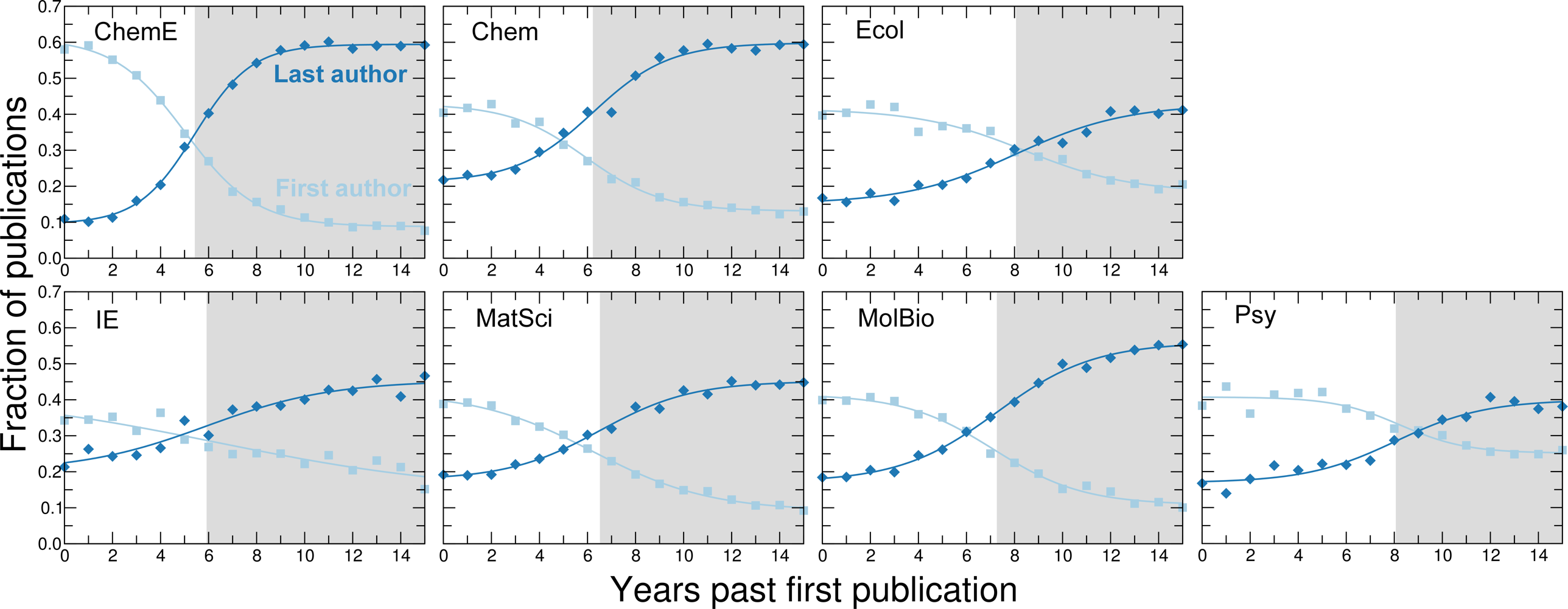

Supplement: Figure S3 — Time to career independence of male faculty members. The fraction of publications authored by male faculty members in which the male faculty member is the last author (blue diamonds) and the fraction of publications in which a faculty member is the first author (azure squares). The blue/azure lines are fits of the data to a generalized logistic function (Methods, Table S11, S12, S13, S14, S15, S16, S17). The grey shaded areas indicate the periods of professional independence for the different disciplines. (TIFF) [file pone.0051332.s003.tiff]

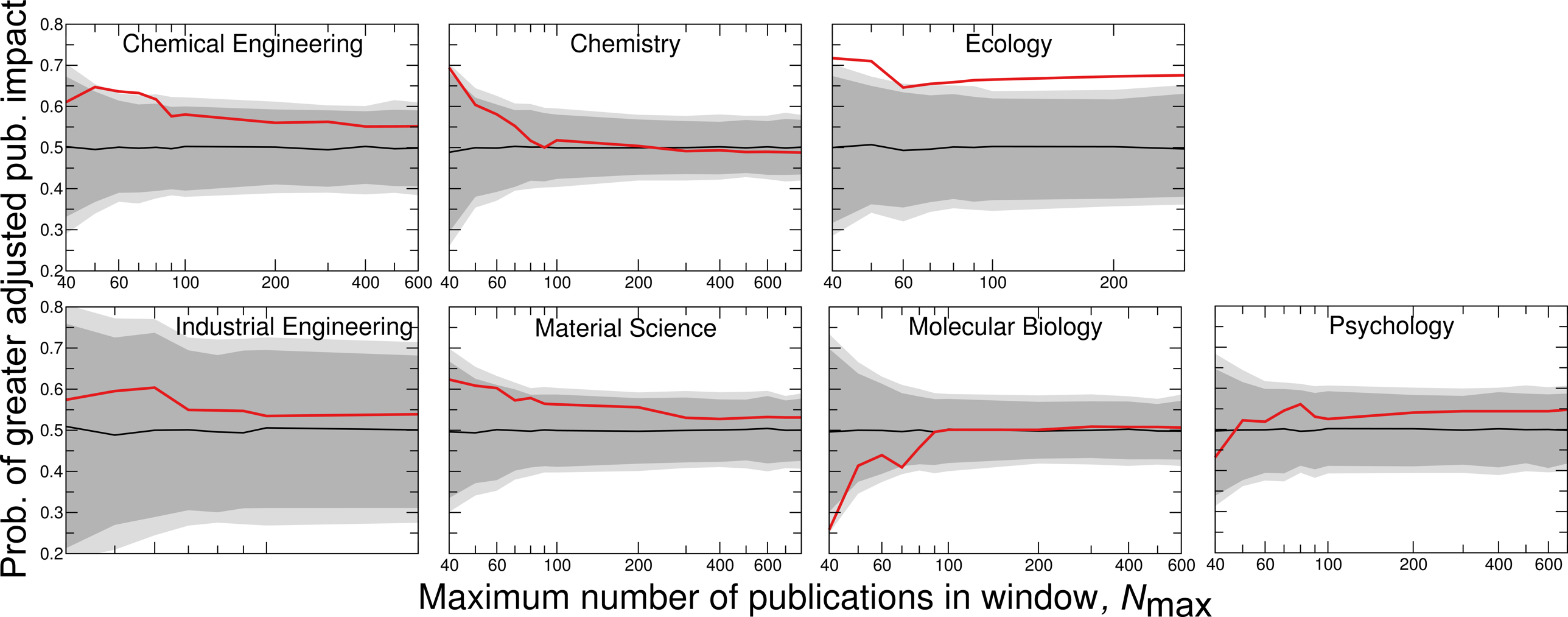

Supplement: Figure S4 — Statistical significance of gender difference in publication impact. Probability that female authors have larger h-index than male authors when accounting for the number of publications. The red line shows the results for windows including authors with at least 30 publications and at most publications. Dark grey areas and light grey areas show the 90% and 95% confidence intervals (see Methods for details). (TIFF) [file pone.0051332.s004.tiff]
