# Supplementary material for: The Possible Role of Resource Requirements and Academic Career-Choice Risk on Gender Differences in Publication Rate and Impact
Source: PLoS One. 2012 Dec 12;7(12):e51332. doi: 10.1371/journal.pone.0051332 (PMC3520933; doi:10.1371/journal.pone.0051332)
Supplement: Table S1 — Gender of faculty in Chemical Engineering departments. (PDF) [file pone.0051332.s005.pdf]

Table S 1. Gender of faculty in Chemical Engineering departments.

Department	Male	Female
California Institute of Technology	7	3
Carnegie Mellon University	19	4
Cornell University	16	1
Georgia Institute of Technology	31	7
Johns Hopkins University	11	2
Massachusetts Institute of Technology	35	4
North Carolina State University	22	2
Northwestern University	14	3
Ohio State University	14	3
Pennsylvania State University	18	4
Princeton University	19	3
Purdue University	23	4
Rensselaer Polytechnic Institute	16	1
Rice University	13	5
Stanford University	17	3
University of California, Berkeley	15	3
University of California, Davis	20	7
University of California, Los Angeles	11	2
University of California, Santa Barbara	17	2
University of Colorado	18	4
University of Delaware	20	4
University of Florida	20	2
University of Illinois at Urbana Champaign	17	3
University of Massachusetts Amherst	15	3
University of Michigan	18	5
University of Minnesota at Minneapolis St. Paul	30	3
University of Notre Dame	17	3
University of Pennsylvania	21	2
University of Texas at Austin	21	2
University of Washington	15	2
University of Wisconsin at Madison	17	2
**Total**	**567**	**98**
