# Supplementary material for: The Possible Role of Resource Requirements and Academic Career-Choice Risk on Gender Differences in Publication Rate and Impact
Source: PLoS One. 2012 Dec 12;7(12):e51332. doi: 10.1371/journal.pone.0051332 (PMC3520933; doi:10.1371/journal.pone.0051332)
Supplement: Table S2 — Gender of faculty in Chemistry departments. (PDF) [file pone.0051332.s006.pdf]

Table S 2. Gender of faculty in Chemistry departments.

Department	Male	Female
Boston University	20	3
California Institute of Technology	21	5
Carnegie Mellon University	24	5
Cornell University	23	3
Duke University	24	3
Emory University	18	2
Georgia Institute of Technology	38	4
Harvard University	20	4
Johns Hopkins University	20	1
Massachusetts Institute of Technology	25	7
North Carolina State University	22	6
Northwestern University	26	4
Ohio State University	33	8
Pennsylvania State University	30	6
Princeton University	17	3
Purdue University	40	15
Rensselaer Polytechnic Institute	19	3
Rice University	19	3
Stanford University	20	3
University of California, Berkeley	50	8
University of California, Davis	28	10
University of California, Los Angeles	43	11
University of California, Santa Barbara	36	4
University of Colorado	43	8
University of Delaware	25	7
University of Florida	38	6
University of Illinois at Urbana Champaign	37	7
University of Massachusetts Amherst	23	4
University of Michigan	36	13
University of Minnesota at Minneapolis St. Paul	36	6
University of Notre Dame	30	5
University of Pennsylvania	28	6
University of Texas at Austin	41	5
University of Washington	31	4
University of Wisconsin at Madison	40	6
**Total**	**1,024**	**198**
