# Supplementary material for: The Possible Role of Resource Requirements and Academic Career-Choice Risk on Gender Differences in Publication Rate and Impact
Source: PLoS One. 2012 Dec 12;7(12):e51332. doi: 10.1371/journal.pone.0051332 (PMC3520933; doi:10.1371/journal.pone.0051332)
Supplement: Table S3 — Gender of faculty in Ecology departments. (PDF) [file pone.0051332.s007.pdf]

Table S 3. Gender of faculty in Ecology departments.

**Department**	**Male**	**Female**
Cornell University	19	6
Duke University	24	7
Harvard University	8	3
Pennsylvania State University	41	17
Princeton University	14	2
Rice University	9	4
Stanford University	12	6
University of California, Berkeley	33	10
University of Chicago	17	3
University of Georgia	20	4
University of Illinois at Urbana Champaign	54	18
University of Michigan	37	14
University of Texas at Austin	11	5
University of Washington	4	2
University of Wisconsin at Madison	26	5
**Total**	**329**	**108**
