# Supplementary material for: The Possible Role of Resource Requirements and Academic Career-Choice Risk on Gender Differences in Publication Rate and Impact
Source: PLoS One. 2012 Dec 12;7(12):e51332. doi: 10.1371/journal.pone.0051332 (PMC3520933; doi:10.1371/journal.pone.0051332)
Supplement: Table S4 — Gender of faculty in Industrial Engineering departments. (PDF) [file pone.0051332.s008.pdf]

Table S 4. Gender of faculty in Industrial Engineering departments.

Department	Male	Female
Cornell University	19	1
Georgia Institute of Technology	47	10
North Carolina State University	19	1
Northwestern University	17	1
Pennsylvania State University	19	3
Purdue University	17	2
Stanford University	27	7
University of California, Berkeley	15	4
University of Florida	14	1
University of Illinois at Urbana Champaign	17	4
University of Michigan	23	4
University of Minnesota at Minneapolis St. Paul	6	2
University of Texas at Austin	6	1
University of Washington	4	4
University of Wisconsin at Madison	12	6
**Total**	**262**	**51**
