# Supplementary material for: The Possible Role of Resource Requirements and Academic Career-Choice Risk on Gender Differences in Publication Rate and Impact
Source: PLoS One. 2012 Dec 12;7(12):e51332. doi: 10.1371/journal.pone.0051332 (PMC3520933; doi:10.1371/journal.pone.0051332)
Supplement: Table S5 — Gender of faculty in Material Science departments. (PDF) [file pone.0051332.s009.pdf]

Table S 5. Gender of faculty in Material Science departments.

Department	Male	Female
Boston University	23	8
California Institute of Technology	8	3
Carnegie Mellon University	17	2
Cornell University	14	3
Duke University	21	4
Georgia Institute of Technology	31	9
Johns Hopkins University	10	2
Massachusetts Institute of Technology	19	6
North Carolina University	12	4
Northwestern University	30	5
Ohio State University	21	4
Pennsylvania State University	20	4
Purdue University	13	2
Rensselaer Polytechnic Institute	9	2
Rice University	14	1
Stanford University	13	1
University of California, Berkeley	18	7
University of California, Los Angeles	17	2
University of California, Santa Barbara	31	3
University of Delaware	12	2
University of Florida	34	6
University of Illinois at Urbana Champaign	19	4
University of Michigan	26	5
University of Pennsylvania	15	5
University of Washington	12	3
University of Wisconsin at Madison	16	4
**Total**	**475**	**101**
