# Supplementary material for: The Possible Role of Resource Requirements and Academic Career-Choice Risk on Gender Differences in Publication Rate and Impact
Source: PLoS One. 2012 Dec 12;7(12):e51332. doi: 10.1371/journal.pone.0051332 (PMC3520933; doi:10.1371/journal.pone.0051332)
Supplement: Table S6 — Gender of faculty in Molecular Biology departments. (PDF) [file pone.0051332.s010.pdf]

Table S 6. Gender of faculty in Molecular Biology departments.

**Department**	**Male**	**Female**
Caltech	12	3
Harvard University	34	10
Johns Hopkins University	24	8
MIT	46	21
Princeton University	34	20
Stanford University	14	10
University of California, Berkeley	32	9
University of California, San Francisco	39	11
University of Texas at Austin	93	30
Washington University in St. Louis	122	35
Yale University	26	12
**Total**	**476**	**169**
