# Supplementary material for: The Possible Role of Resource Requirements and Academic Career-Choice Risk on Gender Differences in Publication Rate and Impact
Source: PLoS One. 2012 Dec 12;7(12):e51332. doi: 10.1371/journal.pone.0051332 (PMC3520933; doi:10.1371/journal.pone.0051332)
Supplement: Table S7 — Gender of faculty in Psychology departments. (PDF) [file pone.0051332.s011.pdf]

Table S 7. Gender of faculty in Psychology departments.

**Department**	**Male**	**Female**
Harvard University	17	9
Princeton University	19	11
Stanford University	21	11
University of California, Berkeley	19	12
University of California, Los Angeles	41	26
University of Illinois, Urbana Champaign	35	21
University of Michigan	65	46
University of Minnesota at Minneapolis	33	8
University of Wisconsin at Madison	18	18
Yale University	14	11
**Total**	**282**	**173**
