# Supplementary material for: The Possible Role of Resource Requirements and Academic Career-Choice Risk on Gender Differences in Publication Rate and Impact
Source: PLoS One. 2012 Dec 12;7(12):e51332. doi: 10.1371/journal.pone.0051332 (PMC3520933; doi:10.1371/journal.pone.0051332)
Supplement: Table S8 — Estimated values of parameters of logistic function for Chemical Engineering data. (PDF) [file pone.0051332.s012.pdf]

Table S 8. Estimated values of parameters of logistic function for Chemical Engineering data.

Gender	Authorship	Parameter estimates			
		A	K	B	M
All	First	0.62 ± 0.01	0.08 ±0.00	0.64 ± 0.03	4.9 ± 0.1
	Last	0.09 ± 0.01	0.60 ±0.01	0.80 ± 0.07	5.4 ± 0.1
Female	First	0.71 ± 0.06	0.00 ± 0.01	0.50 ± 0.08	4.1 ± 0.4
	Last	0.01 ± 0.04	0.63 ± 0.02	0.6 ± 0.1	5.0 ± 0.3
Male	First	0.61 ± 0.01	0.09 ±0.00	0.67 ± 0.04	5.0 ± 0.1
	Last	0.10 ± 0.01	0.59 ±0.00	0.85 ± 0.04	5.4 ± 0.1
