# Supplementary material for: The Possible Role of Resource Requirements and Academic Career-Choice Risk on Gender Differences in Publication Rate and Impact
Source: PLoS One. 2012 Dec 12;7(12):e51332. doi: 10.1371/journal.pone.0051332 (PMC3520933; doi:10.1371/journal.pone.0051332)
Supplement: Table S9 — Estimated values of parameters of logistic function for Chemistry data. (PDF) [file pone.0051332.s013.pdf]

Table S 9. Estimated values of parameters of logistic function for Chemistry data.

Gender	Authorship	Parameter estimates			
		A	K	B	M
All	First	0.43 ± 0.01	0.13 ±0.01	0.64 ± 0.07	5.9 ± 0.2
	Last	0.20 ± 0.01	0.60 ±0.01	0.63 ± 0.08	6.2 ± 0.2
Female	First	0.46 ± 0.01	0.09 ± 0.01	0.67 ± 0.09	5.8 ± 0.2
	Last	0.14 ± 0.02	0.61 ± 0.01	0.7 ± 0.1	6.3 ± 0.2
Male	First	0.43 ± 0.01	0.13 ±0.01	0.63 ± 0.08	5.9 ± 0.2
	Last	0.21 ± 0.02	0.60 ±0.01	0.63 ± 0.09	6.2 ± 0.2
