# Supplementary material for: The Possible Role of Resource Requirements and Academic Career-Choice Risk on Gender Differences in Publication Rate and Impact
Source: PLoS One. 2012 Dec 12;7(12):e51332. doi: 10.1371/journal.pone.0051332 (PMC3520933; doi:10.1371/journal.pone.0051332)
Supplement: Table S10 — Estimated values of parameters of logistic function for Ecology data. (PDF) [file pone.0051332.s014.pdf]

Table S 10. Estimated values of parameters of logistic function for Ecology data.

Gender	Authorship	Parameter estimates			
		A	K	B	M
All	First	0.41 ± 0.01	0.18 ± 0.02	0.5 ± 0.1	8.3 ± 0.5
	Last	0.14 ± 0.01	0.44 ± 0.02	0.5 ± 0.1	8.2 ± 0.3
Female	First	0.41 ± 0.03	0.16 ± 0.03	0.5 ± 0.3	8 ± 1
	Last	0.12 ± 0.03	0.49 ± 0.02	0.6 ± 0.2	8.5 ± 0.6
Male	First	0.41 ± 0.02	0.18 ± 0.02	0.5 ± 0.1	8.4 ± 0.6
	Last	0.15 ± 0.01	0.43 ± 0.02	0.4 ± 0.1	8.1 ± 0.4
