# Supplementary material for: The Possible Role of Resource Requirements and Academic Career-Choice Risk on Gender Differences in Publication Rate and Impact
Source: PLoS One. 2012 Dec 12;7(12):e51332. doi: 10.1371/journal.pone.0051332 (PMC3520933; doi:10.1371/journal.pone.0051332)
Supplement: Table S11 — Estimated values of parameters of logistic function for Industrial Engineering data. (PDF) [file pone.0051332.s015.pdf]

Table S 11. Estimated values of parameters of logistic function for Industrial Engineering data.

Gender	Authorship	Parameter estimates			
		A	K	B	M
All	First	0.37 ± 0.04	0.18 ± 0.05	0.3 ± 0.2	8 ± 2
	Last	0.21 ± 0.02	0.45 ± 0.01	0.4 ± 0.1	6.1 ± 0.6
Female	First	0.39 ± 0.03	0.20 ± 0.04	1 ± 1	9 ± 1
	Last	0.23 ± 0.06	0.44 ± 0.04	0.6 ± 0.6	7 ± 2
Male	First	0.4 ± 0.2	0.1 ± 0.1	0.2 ± 0.3	6 ± 4
	Last	0.20 ± 0.05	0.45 ± 0.02	0.4 ± 0.2	6 ± 1
