# Supplementary material for: The Possible Role of Resource Requirements and Academic Career-Choice Risk on Gender Differences in Publication Rate and Impact
Source: PLoS One. 2012 Dec 12;7(12):e51332. doi: 10.1371/journal.pone.0051332 (PMC3520933; doi:10.1371/journal.pone.0051332)
Supplement: Table S12 — Estimated values of parameters of logistic function for Material Science data. (PDF) [file pone.0051332.s016.pdf]

Table S 12. Estimated values of parameters of logistic function for Material Science data.

Gender	Authorship	Parameter estimates			
		A	K	B	M
All	First	0.43 ± 0.02	0.08 ± 0.01	0.37 ± 0.04	6.0 ± 0.3
	Last	0.17 ± 0.01	0.46 ± 0.01	0.48 ± 0.06	6.6 ± 0.3
Female	First	0.7 ± 0.5	0.0 ± 0.1	0.2 ± 0.2	2 ± 8
	Last	0.10 ± 0.05	0.56 ± 0.05	0.3 ± 0.1	7.7 ± 0.8
Male	First	0.42 ± 0.01	0.09 ± 0.01	0.41 ± 0.04	6.2 ± 0.2
	Last	0.18 ± 0.01	0.45 ± 0.01	0.52 ± 0.07	6.5 ± 0.3
