# Supplementary material for: The Possible Role of Resource Requirements and Academic Career-Choice Risk on Gender Differences in Publication Rate and Impact
Source: PLoS One. 2012 Dec 12;7(12):e51332. doi: 10.1371/journal.pone.0051332 (PMC3520933; doi:10.1371/journal.pone.0051332)
Supplement: Table S13 — Estimated values of parameters of logistic function for Molecular Biology data. (PDF) [file pone.0051332.s017.pdf]

Table S 13. Estimated values of parameters of logistic function for Molecular Biology data.

Gender	Authorship	Parameter estimates			
		A	K	B	M
All	First	0.42 ± 0.01	0.11 ± 0.01	0.54 ± 0.08	7.2 ± 0.3
	Last	0.16 ± 0.01	0.57 ± 0.01	0.48 ± 0.04	7.3 ± 0.2
Female	First	0.42 ± 0.03	0.09 ± 0.03	0.5 ± 0.2	7.6 ± 0.7
	Last	0.14 ± 0.03	0.65 ± 0.03	0.42 ± 0.09	7.8 ± 0.5
Male	First	0.42 ± 0.01	0.11 ± 0.01	0.54 ± 0.07	7.1 ± 0.2
	Last	0.17 ± 0.01	0.56 ± 0.01	0.49 ± 0.04	7.3 ± 0.2
