# Supplementary material for: The Possible Role of Resource Requirements and Academic Career-Choice Risk on Gender Differences in Publication Rate and Impact
Source: PLoS One. 2012 Dec 12;7(12):e51332. doi: 10.1371/journal.pone.0051332 (PMC3520933; doi:10.1371/journal.pone.0051332)
Supplement: Table S14 — Estimated values of parameters of logistic function for Psychology data. (PDF) [file pone.0051332.s018.pdf]

Table S 14. Estimated values of parameters of logistic function for Psychology data.

Gender	Authorship	Parameter estimates			
		A	K	B	M
All	First	0.41 ± 0.01	0.26 ± 0.01	0.9 ± 0.3	8.5 ± 0.5
	Last	0.18 ± 0.01	0.38 ± 0.01	0.7 ± 0.1	8.2 ± 0.3
Female	First	0.43 ± 0.02	0.30 ± 0.02	1.8 ± 2.2	8.5 ± 0.7
	Last	0.19 ± 0.02	0.33 ± 0.02	1.1 ± 0.8	8.0 ± 0.7
Male	First	0.41 ± 0.01	0.25 ± 0.01	0.7 ± 0.3	8.3 ± 0.6
	Last	0.17 ± 0.01	0.40 ± 0.02	0.54 ± 0.15	8.1 ± 0.5
