# Supplementary material for: The Possible Role of Resource Requirements and Academic Career-Choice Risk on Gender Differences in Publication Rate and Impact
Source: PLoS One. 2012 Dec 12;7(12):e51332. doi: 10.1371/journal.pone.0051332 (PMC3520933; doi:10.1371/journal.pone.0051332)
Supplement: Table S15 — Estimated values of parameters of the power law relation between impact and number of publications, . (PDF) [file pone.0051332.s019.pdf]

Table S 15. Estimated values of parameters of the power law relation between impact and number of publications, $h = kn^{\alpha}$.

**Discipline**	α	k
Chemical Engineering	0.58	2.3
Chemistry	0.53	3.3
Ecology	0.56	2.8
Industrial Engineering	0.62	1.5
Material Science	0.59	1.9
Molecular Biology	0.68	2.1
Psychology	0.57	2.8
