# Supplementary material for: The Possible Role of Resource Requirements and Academic Career-Choice Risk on Gender Differences in Publication Rate and Impact
Source: PLoS One. 2012 Dec 12;7(12):e51332. doi: 10.1371/journal.pone.0051332 (PMC3520933; doi:10.1371/journal.pone.0051332)
Supplement: Supporting Information S1. — (PDF) [file pone.0051332.s020.pdf]

Supporting Information

Confidence interval for publication rates. In Fig. S1, we consider the probability that a female author has a publication rate higher than that of a male author as a function of the career stage. Since not all the authors have started their careers in the same year and the publication rate is increasing with time, we consider standard scores relative to career stages in stead of raw publication numbers.

In Fig. S1, we show for each career stage t the quantity $P\left[z_F(t) > z_M(t)\right]$, representing the probability that a female author has a standard score higher than that of a male author at the same stage of her career. We also compute the confidence intervals for these probability values, in the hypothesis that the difference is not due to gender-related reasons. We generate the confidence intervals valid under this hypothesis using a re-sampling method: The populations of females and males are fixed, the values of all standard scores are also fixed, but values of the standard score are randomly reassigned among authors (this is the same as randomly reassigning the genders to authors). For each random configuration, we compute again the probability $P\left[z_F(t) > z_M(t)\right]$ and obtain the confidence intervals by repeating this procedure 1000 times.

Confidence interval for publication impact. In Fig. S4, we consider the probability that a female author has higher publication impact than that of a male author as a function of the number of publications. The analysis is similar to that for publication rates in two aspects: (i) that we use standard scores instead of raw h-indices, and (ii) the method we use to obtain the confidence intervals. The differences are (i) that we consider the h-index and the number of publications instead of the number of publications and the career stage, respectively, and (ii) that the mean and standard deviation are given by Eq. 4.
